# Fatigue Damage Evolution Mechanism of Asphalt Binder Under Variable Stress Repeated Loading

**DOI:** 10.3390/polym17040507

**Published:** 2025-02-15

**Authors:** Weijie Li, Jintao Lin, Weidi Lin, Huayang Yu

**Affiliations:** 1 Guangdong Province Communications Planning & Design Institute Co., Ltd., Guangzhou 510507, China; liweijie@ghdi.cn (W.L.); linweidi@ghdi.cn (W.L.); 2 School of Civil Engineering and Transportation, South China University of Technology, Guangzhou 510640, China; 202321009876@mail.scut.edu.cn

**Keywords:** asphalt binder, variable amplitude stress, damage evolution mechanism

## Abstract

Continuous loading on asphalt pavements induces fatigue damage at the interface between the asphalt binder and aggregate or within the binder itself. The understanding of asphalt’s fatigue response is considered crucial for the prolongation of pavement service life. Variable stress fatigue tests were conducted on asphalt binders, with conditions such as stress amplitude being altered to analyze fatigue performance and life. This study refines asphalt fatigue evaluation systems, introducing a variable stress time sweep test. Modulus recovery after stress changes was revealed through rheological analysis, indicating damage recovery. Fracture surface analysis showed that increased high–stress loadings resulted in reduced edge flow zone width and a flatter surface. Statistical analysis indicated an “exercise effect”, enhancing fatigue life in the second stage. Stress transitions altered fatigue crack paths, surpassing Miner’s linear criterion prediction. The fatigue life curve was accurately fitted using the two–stage life model, affirming its applicability in evaluating variable stress fatigue tests.

## 1. Introduction

Fatigue cracking is a common distress in asphalt road surfaces due to repeated traffic loads. With the increasing traffic volume, many asphalt road surfaces are showing signs of early fatigue cracking. The current road design faces the challenge of ensuring and extending the service life of road surfaces [1]. Fatigue damage in asphalt road surfaces caused by repeated loads mainly occurs at the asphalt binder–aggregate interface (adhesive failure) or within the asphalt binder (cohesive failure) [2]. The traditional fatigue evaluation models exhibit deficiencies and uncertainties in the analysis of fatigue under random loading conditions [3,4,5,6]. Therefore, it is essential to improve the fatigue testing methods for asphalt binders by introducing variable amplitude stresses during load application. Additionally, enhancing the understanding of asphalt binder damage mechanisms based on fatigue accumulation theory is necessary to better align with real traffic conditions [7,8]. Evaluating the fatigue performance of asphalt binders can be performed by analyzing rheological parameters such as the complex modulus and phase angle as they change with the number of loading cycles. However, the underlying micro and macro mechanisms leading to significant variations in these rheological parameters have not been fully understood. Conducting fatigue tests on asphalt binders at ambient temperature reveals complex viscoelastic behavior in the specimens. During testing, various phenomena, including material viscoelasticity, plastic flow, the formation of microdefects/cavities, and macroscopic fracture, can contribute to changes in the mechanical properties of the specimens [9]. In summary, improving the fatigue testing methods of asphalt binders through the introduction of variable amplitude stresses and advancing the understanding of damage mechanisms is crucial for accurately assessing their fatigue performance in real–world traffic conditions.

In the study of asphalt binder fatigue performance, linear amplitude sweep tests are generally conducted to accelerate fatigue testing. These tests involve fixing the test temperature and load frequency while applying linearly increasing amplitudes of load to the specimens [10,11]. The objective is to measure the asphalt material’s fatigue damage threshold. Sabouri et al. conducted linear amplitude sweep tests on asphalt binders under non–linear viscoelastic load control and fatigue tests on asphalt mixtures using a four–point bending beam setup [12]. They analyzed and compared the fatigue performance of binders and mixtures obtained from these two types of tests. The results indicated a close correlation in fatigue performance levels for different types of asphalt in both test methods. This suggests that utilizing linear amplitude sweep tests for binders can effectively predict the corresponding fatigue performance of asphalt mixtures [13,14].

In a similar vein, Yu et al. performed linear amplitude sweep tests on warm–mix rubberized asphalt. The findings demonstrated that the addition of rubber powder and organic warm mix additives can significantly enhance the fatigue resistance of the asphalt material. Furthermore, the fatigue life predictions from linear amplitude sweep tests showed good correlation with fatigue test results for asphalt mortar and asphalt mixtures [15].

While linear amplitude sweep tests can effectively reduce the time required for assessing asphalt fatigue performance, they have limitations in terms of analyzing test parameters. These tests yield fixed cumulative damage results and do not provide insights into the continuous evolution of fatigue characteristics in asphalt binders under varying load cycles [16]. This makes it challenging to compare the evolving damage processes under different loading modes. Consequently, this study employs time sweep tests to facilitate the continuous observation of this process, applying variable amplitude stress combinations to asphalt binders to observe the fatigue response during stress level transitions [17,18].

In fatigue testing for various engineering materials, the Miner linear cumulative damage model is the most commonly used model to assess material damage accumulation [19]. This model treats the damage from different loads as equivalent and assumes that the material’s damage accumulation process is linear. When asphalt binders are subjected to alternating stresses or strains of varying magnitudes, changes in load amplitudes can influence the form and path of specimen damage evolution [20,21]. In actual road applications, the number and sequence of varying–sized axle loads are continually changing. The asphalt pavement life calculation method based on the Miner linear fatigue damage accumulation criterion neglects the influence of the load history on asphalt fatigue degradation behavior, resulting in significant discrepancies between predicted and actual values [22,23]. Tanaka, based on fatigue test results on nickel–chromium alloy wire specimens, modified the Miner criterion: ∑*ni*/*Ni* = *a*, where *ni* is the actual number of fatigue cycles at each stress level, while *Ni* is the theoretical fatigue life at that stress level and a is a constant related to the material properties and can be determined through fatigue testing [24].

Hintz and others conducted time sweep tests on asphalt binders, combined with digital visualization techniques, to study crack propagation trends in asphalt samples. They performed digital visualizations of the fracture surface of binder specimens after the tests, determining crack propagation paths and the effective radius of the remaining specimen. They found that crack propagation predictions based on load resistance measurements were consistent with direct measurements based on digital visualization [25]. Zhang and colleagues subjected asphalt binders to time sweep tests under both non–linear viscoelastic (NLVE) and linear viscoelastic (LVE) stress conditions. After the tests, they used an optical camera to capture images of the fracture surfaces between the upper and lower parallel plates of the instrument. They applied image processing techniques to analyze the various textures on the fracture surface, summarizing the stages of damage corresponding to different types of textures [9]. Hintz and others, by combining fracture surface images with rheological performance parameters output from a dynamic shear rheometer (DSR), confirmed that asphalt binders undergo two types of fatigue damage during time sweep tests: elastic damage and plastic flow damage. Under light loads, asphalt samples are predominantly subject to elastic damage, whereas heavy loads lead to plastic flow damage [26]. During the process of elastic damage, the asphalt binder’s phase angle remains relatively constant, while the complex shear modulus steadily decreases [27]. During the process of plastic flow damage, noticeable changes are observed in the slopes of the curves corresponding to complex shear modulus and phase angle. The study also observed that the contour of the fracture surface is concave rather than flat, attributing this phenomenon to the normal stress applied by instrument vibration during loading [28]. In summary, the research highlights the complexity of fatigue damage in asphalt binders and the need for comprehensive testing and analysis methods to understand and predict the material’s performance under varying load conditions.

In this study, time sweep tests were conducted on asphalt binders under different loading modes. A dynamic shear rheometer (DSR) was utilized to apply cyclic stress to the binder specimens, and the changes in their rheological properties and energy dissipation characteristics were studied. This information served as the basis for evaluating the materials’ fatigue resistance. By adjusting parameters such as loading cycles, loading sequences, and load amplitudes, this study designed constant stress fatigue tests and two–stage variable amplitude stress fatigue tests for binder materials, and then conducted a comparative analysis. In the time sweep tests, cylindrical specimens were subjected to periodic shear loading, resulting in circumferential cracks that initiated at the specimen’s periphery and propagated inwards. Fracture surface images of the binder specimens collected after the tests were used to characterize the distinctive features of the fracture surfaces under different loading modes. Combining the knowledge of damage mechanics, this study analyzed the differences in the damage evolution processes under various loading modes. This research connected the features of the fracture surfaces to the different stages in the rheological performance curves of the binders, thus revealing the transition from fatigue cracking to plastic flow damage, and ultimately, the occurrence of failure in the asphalt specimens. This study introduces a novel testing approach that addresses the limitations of traditional fatigue evaluation models under random loading conditions, enhancing the reliability of fatigue life predictions and providing deeper insights into the damage mechanisms of asphalt binders.

## 2. Materials and Testing Methods

### 2.1. Asphalt Binders

**Figure 1:**
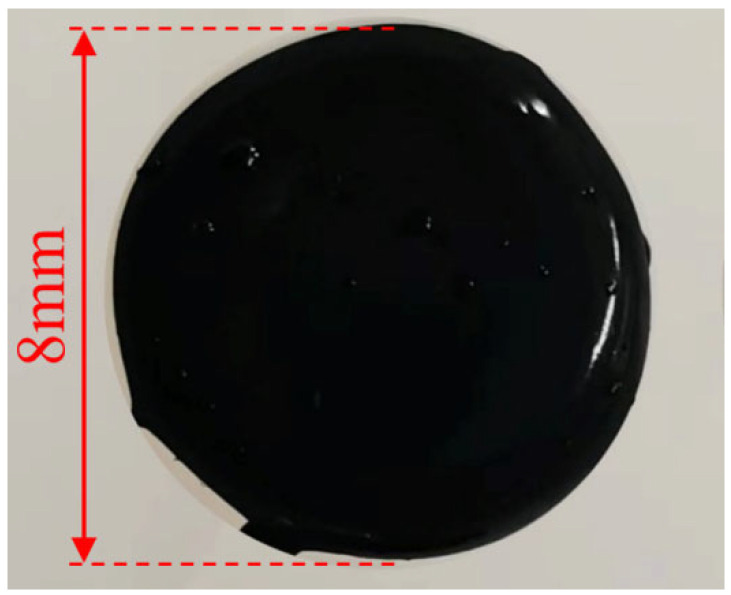
Preformed asphalt binder specimens.

In this study, three types of asphalt binders were selected, including two base asphalt binders, labeled as Pen70–80 and Pen60–70, as well as one SBS–modified asphalt, labeled as SBS. All these asphalt binders are manufactured by the Shell Company, and their key technical specifications are presented in Table 1. Short–term aging was applied to all the asphalt binders before sample preparation to simulate their condition after construction. Before sample preparation, the hot asphalt was stirred with a glass rod for approximately 30 s to ensure uniform properties and remove air bubbles. The stirred asphalt binders were poured into silicone molds to create asphalt specimens, and the precast binder material specimens obtained after demolding are depicted in Figure 1.

### 2.2. Stress Sweep Test

**Figure 2:**
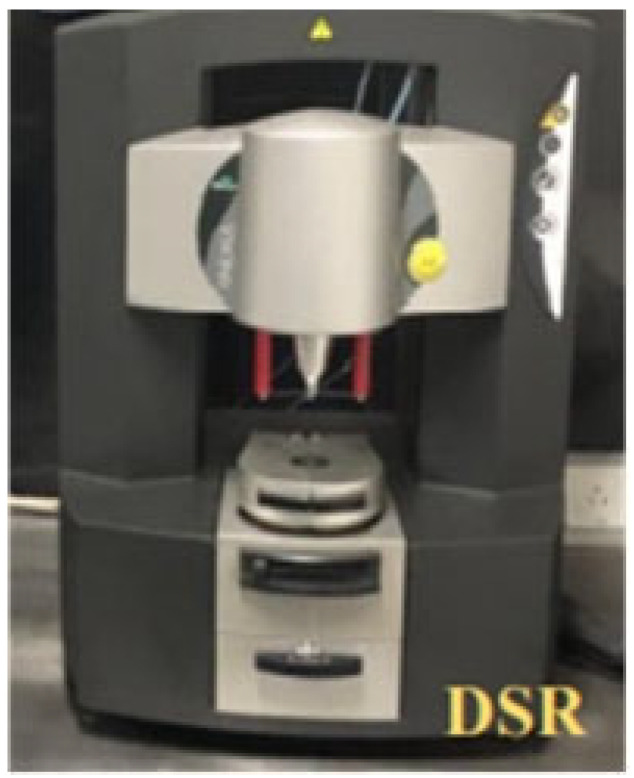
The DSR testing setup.

Both stress sweep and time sweep tests in this study were conducted using the Kinexus dynamic shear rheometer from Malvern Instruments Ltd., Malvern, UK. The testing setup is shown in Figure 2. The parallel plate system of the rheometer has a diameter of 8 mm and a gap size of 2 mm. The test temperature and frequency for all samples were set at 25 °C and 10 Hz, respectively, with raw data collected every second, corresponding to every 10 loading cycles. After each test, the temperature was lowered to 3 °C and held for 10 min to prevent damage to the fracture surface when separating the upper and lower plates. The lower plate was then removed, and images of the fracture surface on the plate were captured using an optical camera for further analysis.

The purpose of the stress sweep test was to determine the linear viscoelastic region of the asphalt binder. During the stress sweep test, a logarithmically increasing shear stress was continuously applied to the asphalt samples within a stress range of 10 kPa to 1000 kPa. According to ASTM D7175–15 specifications, the yield stress for each asphalt binder in this study was defined as the shear stress corresponding to a 10% decrease in the residual complex shear modulus during the stress sweep process compared to the initial complex shear modulus. To ensure accurate test results, each asphalt binder was subjected to three or more repeated tests, and the obtained yield stress values are listed in Table 2.

### 2.3. Stress–Controlled Time Sweep Test Procedure

To obtain a comprehensive fatigue damage process for the asphalt samples, this study employed reaching the peak phase angle as the criterion for the fatigue failure of the asphalt binder specimens [27]. For each set of test conditions, at least two repeated tests were conducted until the data variability between the two sets was less than 10%. Subsequently, suitable stress combinations were selected to perform a two–stage variable amplitude time sweep (VATS) test.

Different asphalt binder types had VATS test procedures developed based on different damage levels in the first stage, as shown in Table 3. After subjecting the asphalt specimens to n1 cycles of load τ_1_ in the first stage, the application of the second–stage load τ_2_ was continuous until the phase angle of the specimen reached its peak, upon which the test was stopped to obtain the measured value of the specimen’s second–stage fatigue life. This study conducted three or more repeated tests for each loading pattern in the time sweep, ensuring that the error in the measured fatigue life of the specimens was kept within 10%.

**Figure 3:**
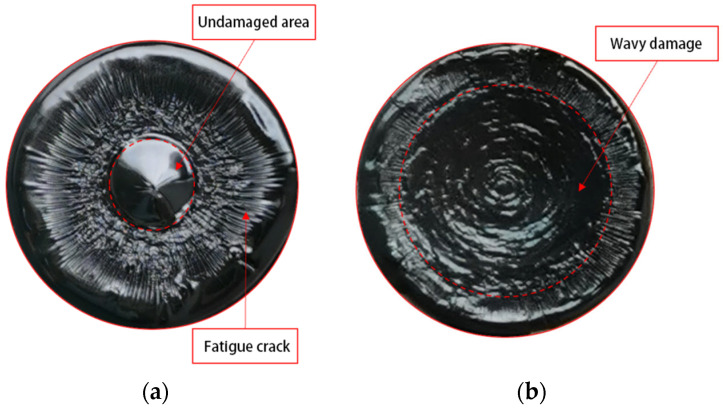
Fracture surface morphologies of Pen70–80 asphalt binder at different stress levels: comparison between 100 kPa (**a**) and 300 kPa (**b**).

To provide a more scientific explanation of the fatigue damage process under variable amplitude stress, Zhang and others introduced the concept of effective stress, defining it as the ratio of the load endured by the sample to the effective area [9]. At the end of the test, after lowering the temperature to 3 °C and allowing a period of rest, the upper rotor is raised. If the asphalt sample has not been completely destroyed, a flat and smooth surface can be observed in the central area of the asphalt sample. This smooth area is the part of the sample that remains intact and is created when the sample is pulled apart by the instrument. It corresponds to the region where the sample can still withstand a certain number of load cycles after the test. Typically, under lower LVE stress, the sample does not necessarily break completely at the end of the constant stress time sweep. An undamaged area can exist inside the sample. For example, in the case of the Pen70–80 asphalt binder subjected to 100 kPa stress until fatigue failure, an optical camera captures the overhead view of the lower chassis (as shown in Figure 3a). Specifically, under NLVE stress, plastic flow damage predominates during the fatigue failure process (Figure 3b). With the transition from LVE stress to NLVE stress at the end of the test, the sample fracture surface obtained may have very few or even no cracks. Similarly, when the load transitions from LVE stress to NLVE stress, the sample’s fracture surface is expected to display both crack damage and wave–like damage (Figure 3a).

### 2.4. The Two–Stage Fatigue Life Model

The fatigue life of the asphalt binder under two–stage variable amplitude loading was compared with the predictions made using the Miner linear criterion, revealing significant discrepancies between the two. The non–linearity of the fatigue damage accumulation process and the transition between different types of damage in the second stage contribute to the challenges in accurately predicting the fatigue life. Zhang and Oeser proposed a two–stage fatigue life model for NLVE–LVE loading patterns, referred to as the ORZ model:

(1) $${\left(\frac{n_{1}}{N_{f1}}\right)}^{a}+\left(\frac{n_{2}}{N_{f2}}\right)=1$$

where *n*_1_ represents the number of stress cycles in the first stage, *n*_2_ is the number of stress cycles in the second stage, *N_f_*_1_ is the fatigue life of the sample under the action of stress in the first stage alone, *N_f_*_2_ is the fatigue life of the sample under the action of stress in the second stage alone, and “*a*” is a parameter that reflects the deviation of experimental values from Miner’s criterion predictions. The value of “*a*” is mainly influenced by factors such as asphalt binder type, load magnitude, loading sequence, and test temperature.

This model establishes a relationship between the two–stage fatigue life under stress control with different amplitudes. It can be used to predict the remaining fatigue life of the second stage after any number of stress cycles in the first stage using limited fatigue test data.

The parameter “*a*” is fitted by the root mean square error (RMSE).

(2) $$RMSE=\sqrt{\frac{\sum_{J=1}^{J}\left({\hat{y}}_{j}+y_{j}\right)}{J}}$$

In the provided formula, RMSE represents the root mean square error, *J* stands for the number of load cycles, and ${\hat{y}}_{j}$ denotes the model’s predicted values, while *y_j_* corresponds to the experimentally measured values.

## 3. Results and Discussion

### 3.1. Fracture Surface Morphology Analysis

In the field of damage mechanics, the fracture surface serves as an effective means to reveal the material’s damage mechanisms and pathways of propagation. The term “fracture surface” in this study refers to the surface texture presented by asphalt specimens during fatigue failure in dynamic shear rheometer tests. It is also referred to as the fracture mode, fracture morphology, or failure morphology. In time sweep tests, the fatigue damage propagation path of cylindrical asphalt specimens extends from the outer edges towards the center. During this process, the effective load–bearing area of the specimen continuously decreases until fracture failure occurs, eventually leading to the almost complete separation of the asphalt specimens between the upper and lower plates. After the experiments, surface images of the asphalt specimens on the lower plate were directly captured, revealing an approximately circular fracture surface. (Note that initially, the instability of material flow at the specimen’s edges can result in irregular protrusions at the edges of the fracture surface, causing the fracture surface not to be perfectly circular). Different areas on the fracture surface exhibit varying morphologies, which are associated with the types of fatigue damage observed in each stage of the experiments. Each damage zone appears as a circular ring with the center of the fracture surface as its focal point.

#### 3.1.1. Fracture Surface Morphology Analysis Under Constant Stress

**Figure 4:**
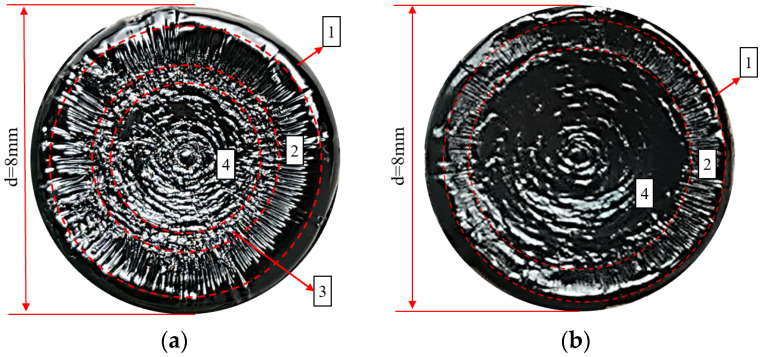
Fracture surface images after constant stress–time scanning: (**a**) at 150 kPa and (**b**) at 300 kPa.

Figure 4 shows the fracture surfaces of Pen70–80 asphalt specimens after loading with 150 kPa and 300 kPa loads. To make the boundaries of the different damage areas more distinct, the images were sharpened (sharpened by 50%) and marked with red dashed lines. It is evident that there are distinct differences in the damage process of the specimens under LVE (low–velocity elastic) and NLVE (non–low–velocity elastic) stresses.

In Figure 4, Area 1 represents the edge flow region, where the unstable flow of the asphalt specimen’s edge occurs during the initial stages of loading. Area 2 is the cracking zone, where fatigue cracks start to form and propagate radially from the outside to the inside. Area 3 is the transition zone where crack damage transforms into plastic flow damage. As the test progresses, the continuous expansion of fatigue cracks in the asphalt specimen leads to a reduction in the effective load–bearing area and a corresponding increase in effective stress until it exceeds the structural strength of the specimen. This results in a transition from crack–dominated damage to plastic flow, causing the asphalt to flow into the crack valleys, gradually filling them and creating an irregular morphology, which we label as the transition zone. Area 4 is the wavy damage zone, characterized by plastic flow damage being the dominant mode of failure.

It can be observed that under LVE stress, the asphalt specimen primarily experiences fatigue damage with a wider crack zone and a smaller wavy damage area. In contrast, under NLVE stress, flow damage dominates, leading to a noticeably narrower crack zone and a larger wavy damage area, with no clear transition zone observed.

#### 3.1.2. The Evolution of Fracture Surface Damage Zone Morphology and Width Under Variable Stress

**Figure 5:**
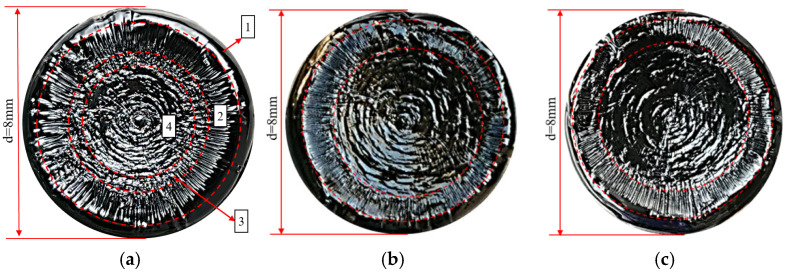

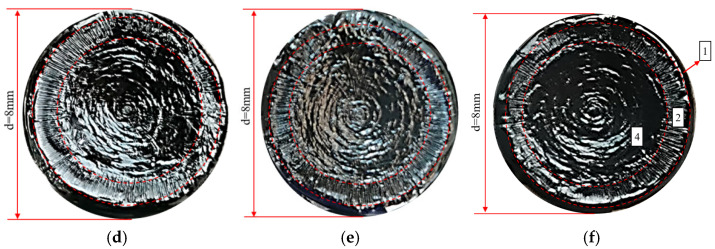
Fracture surface images obtained from different loading modes in variable amplitude time scanning tests: (**a**) 150 kPa; (**b**) 150–300–4; (**c**) 150–300–3; (**d**) 150–300–2; (**e**) 150–300–1; and (**f**) 300 kPa.

The Pen70–80 asphalt specimens were subjected to constant stress–time scanning at both 150 kPa and 300 kPa, as well as variable stress–time scanning from 150 kPa to 300 kPa. After the tests were completed, fracture surface images of the asphalt specimens were acquired and sharpened by 50%, as shown in Figure 5.

Subsequently, the widths of different types of damage zones were measured. Due to the influence of the testing instrument’s precision, the geometric centers of the damage zones do not strictly align with the center of the fracture surface. Therefore, a cross–shaped sampling method was employed, selecting eight evenly distributed points within each damage zone. The width data at each of these points were measured, and the average width was then calculated to determine the accurate width of the damage zone.

**Figure 6:**
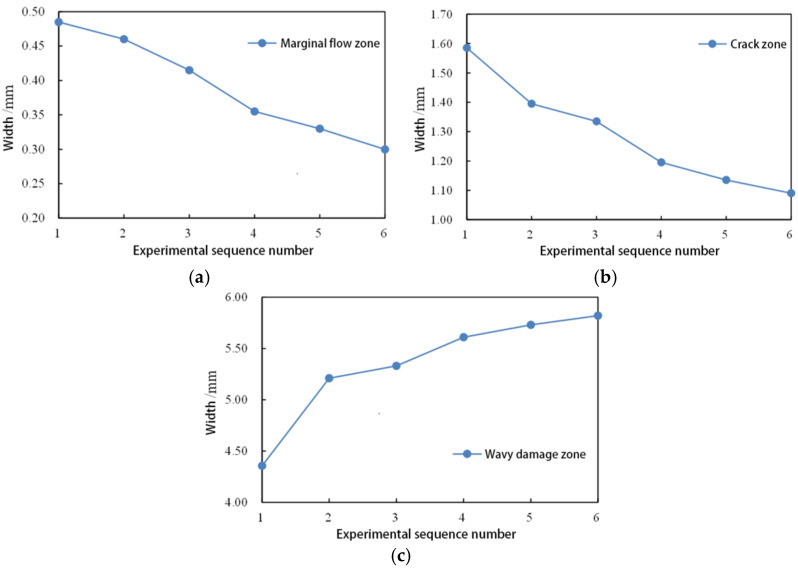
Variation in width of different damage zones on the fracture surface in time–scanning tests under 150–300 loading modes: (**a**) edge flow zone; (**b**) crack zone; and (**c**) wavy damage zone. (Note: test numbers 1–6 correspond to the following loading modes in sequence: 150 kPa constant stress scanning, 150–300–4, 150–300–3, 150–300–2, 150–300–1, and 300 kPa constant stress scanning).

To visually represent the impact of changing the first–stage load on the evolution of asphalt damage, width variation curves for different damage zones were plotted, as depicted in Figure 6.

From Figure 5, it is noticeable that in the 150 kPa constant stress scanning test, there is a clear transition zone on the fracture surface of the asphalt, whereas in the 150–300 variable amplitude time scanning, no transition zone is observed. This suggests that, in the case of applying NLVE stress in the second stage, the type of damage transitions abruptly from fatigue cracks to plastic flow damage. Examining the variation curves for different regions in Figure 6, it is evident that the width of the edge flow zone on the fracture surface gradually decreases from test numbers 1 to 6.

Upon comparing the data from the tests, it is revealed that, even though the normal stress acting on the asphalt binder is lower under LVE stress compared to NLVE stress, the loading duration is significantly longer for LVE stress. This prolonged loading process amplifies the influence of the normal stress. Therefore, the width of the edge flow zone on the fracture surface is noticeably larger in the case of the 150 kPa constant stress scan than in the 300 kPa constant stress scan. Furthermore, the normal stress results in a concave fracture surface after the asphalt specimen undergoes fatigue failure. After the fatigue test with NLVE stress is completed, the fracture surface of the asphalt binder appears flatter along its longitudinal axis compared to the fracture surface under LVE stress. This is because, under LVE stress, while the normal stress is relatively lower, the longer loading time results in a wider edge flow zone on the fracture surface, leading to a noticeable concave shape. In contrast, under NLVE stress, despite the higher normal stress, the shorter loading duration reduces the influence of the normal stress, resulting in a narrower edge flow zone and a flatter fracture surface.

The width variation in different damage zones shown in Figure 6 indicates that reducing the frequency of LVE stress applications in the first stage leads to a gradual decrease in the width of the fatigue crack zone on the fracture surface and an increase in the area of the wavy damage zone.

### 3.2. Analysis of Damage Evolution Law Based on Sample Fatigue Life

#### 3.2.1. Fatigue Life of Specimens Under Two Types of Variable Stresses

The analysis of the fatigue life data for Pen70–80 asphalt under LVE–NLVE loading modes at various stages is shown in Table 4. These tables show the statistical results of fatigue life for four different loading modes: 150–300, 150–400, 100–300, and 100–400.

**Figure 7:**
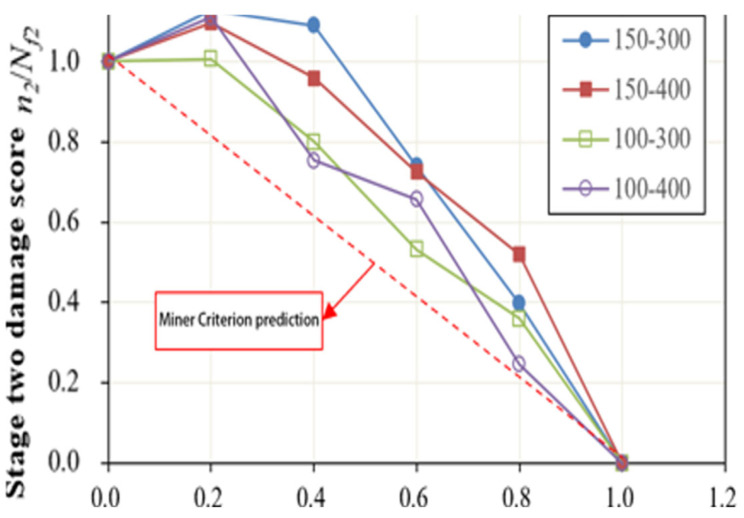
Fatigue life curve of asphalt Pen70–80 under LVE–NLVE loading mode.

Based on the experimental data presented in Table 4, fatigue life curves for the second stage were plotted with the first–stage damage score as the horizontal axis and the second–stage damage score as the vertical axis, as illustrated in Figure 7. Comparing these fatigue life curves for the four different LVE–NLVE loading modes, the results provide insights into how altering the stress levels in either the first or second stage influences the cumulative damage outcome of the specimens.

In the four LVE–NLVE loading modes, it was observed that after the first–stage cyclic loading with a number of cycles equal to 0.2*N_f_*_1_, the remaining fatigue life of the asphalt specimens in the second stage, denoted as *n*_2_, exceeded the fatigue life *N_f_*_2_ obtained in NLVE constant stress scanning tests. In other words, *n*_2_ > *N_f_*_2_. This phenomenon cannot be adequately explained by the traditional Miner’s linear damage rule. As the number of cycles in the first–stage loading was increased to 0.4*N_f_*_1_, only the 150–300 stress combination showed *n*_2_ exceeding *N_f_*_2_. However, as the number of cycles in the first stage increased further, this phenomenon disappeared for all stress combinations. This suggests that the specimens, after undergoing a relatively low number of LVE stress cycles (below a certain critical threshold), experience an increase in the fatigue life of the second stage when subjected to higher amplitude NLVE stress cycles. When the number of cycles of LVE stress in the first stage exceeds a critical value, for instance, when the first–stage cycle numbers are 0.6 *N_f_*_1_ and 0.8*N_f_*_1_, the specimens begin to exhibit significant damage in the first stage, and the remaining fatigue life in the second stage rapidly decreases. The “training effect” is no longer significant.

Furthermore, all data points in Figure 7 lie above the predicted values according to Miner’s rule. This implies that the sum of the two–stage fatigue life obtained in the four LVE–NLVE loading modes is consistently greater than 1, meaning *n*_1_/*N_f_*_1_ + *n*_2_/*N_f_*_2_ > 1. This exceeds the fatigue life predicted by Miner’s linear damage accumulation rule. It indicates that the fatigue design method based on Miner’s rule underestimates the fatigue performance of asphalt binders when subjected to LVE–NLVE combined loading. While this approach yields conservative results, it leads to increased resource consumption and might not be cost–effective.

**Figure 8:**
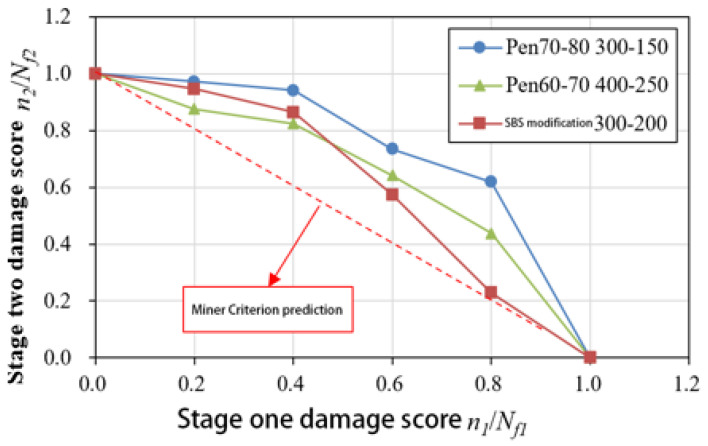
Fatigue life curve of NLVE LVE loading mode.

A fatigue life curve for the three asphalt binder specimens under NLVE–LVE loading modes is depicted in Figure 8. In this testing procedure, the number of cycles with increasing stress *τ*_1_ during the first stage is incrementally raised. The results show that the remaining fatigue life *n*_2_ of the specimens in the second stage consistently falls short of the fatigue life *N_f_*_2_ obtained in the LVE constant stress scanning tests. This indicates that the asphalt binders did not exhibit the “training effect” when subjected to higher amplitude NLVE stress during the first stage.

This absence of a training effect can be attributed to the fact that stress levels exceeding the linear viscoelastic region exert irreversible damage on the asphalt binders in the initial loading stages. When the extent of damage surpasses the level of enhancement, the accumulated damage in the asphalt binders continues to increase, and the accumulation rate gradually accelerates. At this point, the asphalt specimens have undergone degradation, resulting in a total two–stage fatigue life that is less than the fatigue life when subjected to low–amplitude stress alone.

However, even under this mode, the sum of the two–stage fatigue life still exceeds the prediction of Miner’s rule, primarily due to the residual stress from the first–stage NLVE stress. When low–amplitude stress is applied, the specimens exhibit a residual stress state inherited from the previous high–amplitude stress loading. This residual stress is gradually dissipated during the low–amplitude loading process, which is accompanied by the partial recovery of the complex shear modulus. Such recovery reflects the material’s ability to reorganize internal structures, which helps mitigate the effects of prior damage. Furthermore, during the transition between stress levels, the damage evolution path undergoes noticeable changes, demonstrating a lag in damage accumulation as the stress shifts from high to low. The varying degrees of complex shear modulus recovery observed at this stage provide evidence of the material’s intrinsic capacity for damage delay. This phenomenon, commonly referred to as the “exercise effect”, can be attributed to the asphalt binder’s ability to redistribute residual stresses and recover from microcrack–induced modulus decay. In particular, low–amplitude stress may play a critical role in facilitating the healing of microcracks by promoting molecular realignment within the binder matrix. This process contributes to stress homogenization across the material, effectively delaying further damage accumulation and improving the fatigue resistance during the second stage of loading.

Nevertheless, it is important to note that different results have been obtained in other studies using variable amplitude stress fatigue tests. In practice, the second–stage damage score is not fixed, as the process of damage evolution is quite complex and can be influenced by factors such as material microstructure, loading sequence, and the difference in stress amplitude between the two stages. The greater the difference in stress amplitude between the two stages, the more significant the impact of the stress from the first stage on the cumulative damage in the second stage.

**Figure 9:**
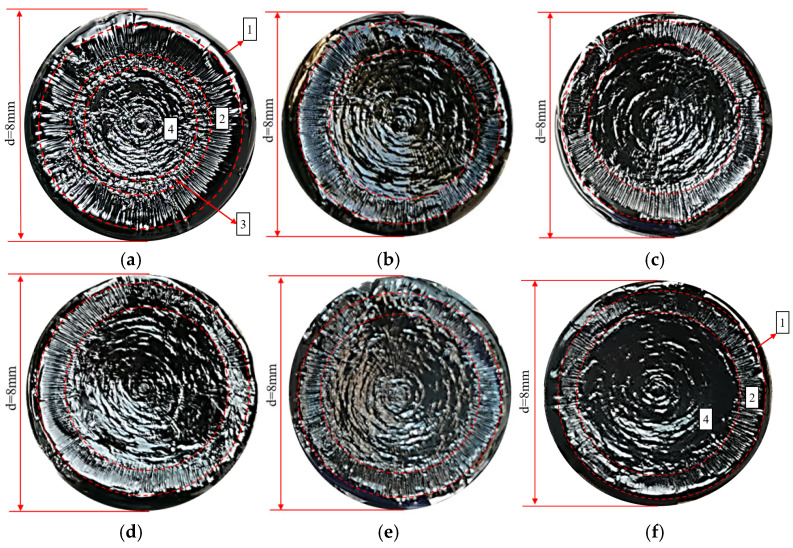
Fracture surface images obtained from different loading modes with variable amplitude time scanning experiments: (**a**) 150 kPa; (**b**) 150–300–4; (**c**) 150–300–3; (**d**) 150–300–2; (**e**) 150–300–1; and (**f**) 300 kPa.

Examining the fracture surface images of the asphalt specimens in Figure 9 provides insights into the fatigue crack evolution process. During loading, the expansion of fatigue cracks leads to a gradual reduction in the effective load–bearing area of the specimen, resulting in an increase in effective stress. Once this effective stress surpasses the structural strength of the asphalt specimen, it experiences overloading, and the mode of damage evolution shifts from fatigue crack propagation to plastic flow. The transition in the mode of damage evolution results in a delay in damage accumulation. Research on the fatigue performance of central crack panels has shown that after undergoing overloading, the fatigue crack propagation path in the material changes, and the rate of crack propagation rapidly decreases. This phenomenon is known as the fatigue crack propagation delay effect and, to some extent, contributes to enhancing the material’s fatigue resistance and extending its fatigue life [24]. The mechanism underlying the generation of the delay effect can be explained using the plastic–induced crack closure mechanism. This mechanism posits that the plastic damage zone generated after overloading experiences compression from the elastic material outside the plastic region, creating a residual stress field within the plastic region. After overloading, the extension of the plastic residual deformation zone at the crack tip in the asphalt binder increases, leading to the continuous expansion of the residual compression stress field. This, in turn, elevates the required opening stress for crack extension, thereby prolonging the time it takes for the crack to propagate. After this transitional period, the plastic deformation caused by cyclic loading initiates the re–accumulation of fatigue damage. The extent of crack propagation during the transitional period depends on the ratio of the two–stage loading in the variable amplitude stress. After applying overloading, the rate of crack propagation immediately decreases until it stops expanding, after which the specimen begins to exhibit plastic flow damage. This transitional phase of crack propagation plays a crucial role in understanding how asphalt materials respond to cyclic loading and how the fatigue resistance of the material is affected by changes in loading conditions, such as the presence of overloading or the transition from fatigue crack propagation to plastic flow damage.

#### 3.2.2. Analysis of Two–Stage Fatigue Life Model

This study aims to validate the applicability of the model under LVE–NLVE loading modes. Data obtained from six different loading modes for Pen70–80 asphalt, including 100–300, 100–400, 100–500, 150–300, 150–400, and 150–500, were selected for validation. The first–stage stresses for the chosen loading modes are 100 kPa and 150 kPa, each corresponding to three gradients of second–stage stress. The values of parameter *a* were determined based on the minimum root mean square error for each loading mode, as listed in Table 5.

The trend in the variation of parameter *a* values for each loading mode in Table 5 reflects the influence of the stress levels in each stage on the fatigue life of the asphalt specimens. A higher value of parameter *a* indicates a greater deviation of the fitted curve from Miner’s rule prediction, resulting in lower accuracy when predicting fatigue life using Miner’s rule for that loading mode. According to Table 5, it is observed that, for loading modes where the same level of stress is applied in the second stage, the *a* values are consistently higher for loading modes with 150 kPa stress applied in the first stage compared to those with 100 kPa stress. When the first–stage stress amplitude is held constant and the second–stage stress is increased, the *a* values also increase simultaneously. A clear pattern emerges: reducing the second–stage stress or decreasing the stress difference between the two stages leads to an increase in the *a* value, indicating a greater deviation between the experimental results and predictions.

**Figure 10:**
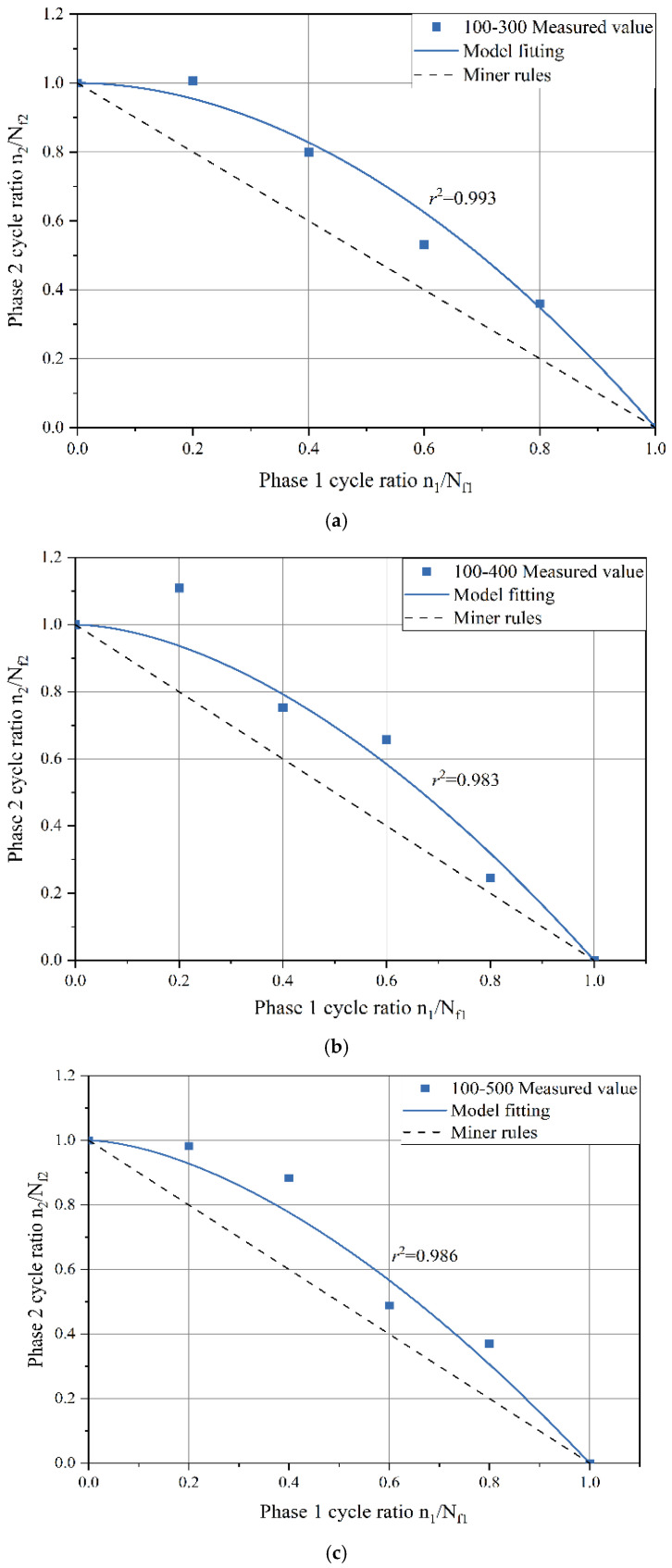
Second–stage residual life fitting curve (first–stage stress 100 kPa): (**a**) 100–300 Measured value; (**b**) 100–400 Measured value; (**c**) 100–500 Measured value.

By substituting the *a* values from Table 5 into the two–stage fatigue life model, fitted curves for the remaining specimen life in the second stage were obtained for each loading mode, as shown in Figure 9 and Figure 10. The figures display the coefficient of determination (*r*^2^) between the fitted curves and the experimentally measured values. Higher *r*^2^ values (*r*^2^ > 0.95) indicate a higher level of accuracy in the model’s predictions, demonstrating that the model can effectively predict the second–stage damage score (i.e., remaining fatigue life) for asphalt binders under LVE–NLVE loading modes with a high degree of precision.

**Figure 11:**
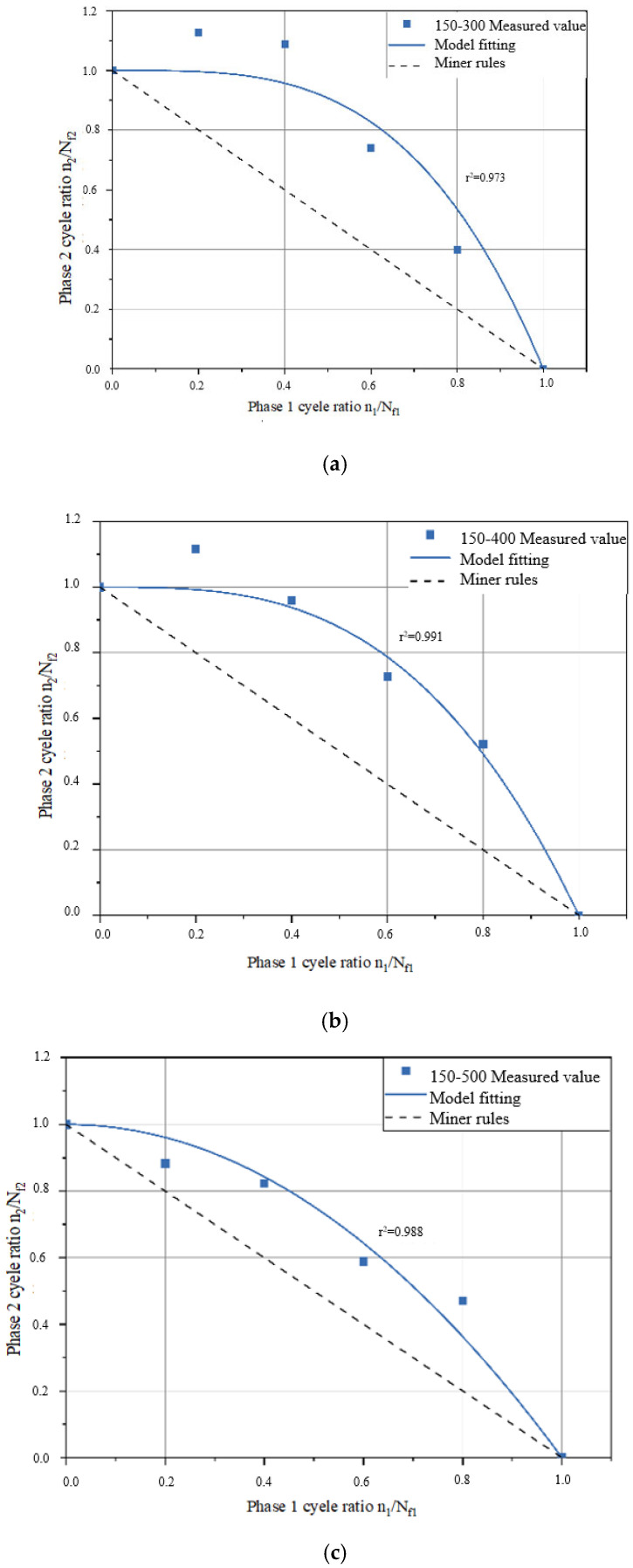
Fitting curve of residual life in the second stage (first–stage stress 150 kPa). (**a**) 150–300 Measured value; (**b**) 150–400 Measured value; (**c**) 150–500 Measured value.

In Figure 10, corresponding to the loading mode where the first–stage stress is consistently 100 kPa, it can be observed that as the second–stage stress gradually increases, the curvature of the fitted curve gradually decreases. Figure 11 exhibits the same pattern. Combining the findings in Table 5, it becomes evident that with higher second–stage stress levels, the *a* values also increase, signifying a reduced deviation from Miner’s rule predictions. This is because, in the second stage of loading, when high stress amplitudes exceed a certain range, the fatigue damage process is dominated by the high stress, and the influence of the “training effect” from the low–stress first stage diminishes. This ultimately results in reduced fatigue life.

The measured values and model predictions for the second–stage cyclic ratio in each loading mode are presented in Table 6, allowing for a comparison of the differences between the two. In the 100–400 loading mode, when the first–stage load cycle ratio (i.e., damage score) is 0.2, the difference between the ORZ model predictions and the experimental measurements is notably larger than when the damage scores are 0.4, 0.6, and 0.8. Furthermore, for test numbers 150–300–1, 150–300–2, and 150–400–1, the differences between the predicted values and the measured values all exceed 0.1.

In the LVE–NLVE loading modes, when the first–stage damage score is 0.2 or 0.4, the second–stage damage score often exceeds 1. This means that the fatigue life in the second stage has already exceeded the fatigue life under the influence of high stress in the second stage, surpassing the applicability range of the ORZ model. Under the LVE–NLVE loading modes, the model is better suited for predicting the remaining fatigue life of specimens when the first–stage damage score is relatively high. By applying the ORZ model to the LVE–NLVE loading modes, it is possible to quantitatively analyze the impact of the magnitude of NLVE stress and the number of load cycles in each stage on the two–stage fatigue life of asphalt binders.

**Figure 12:**
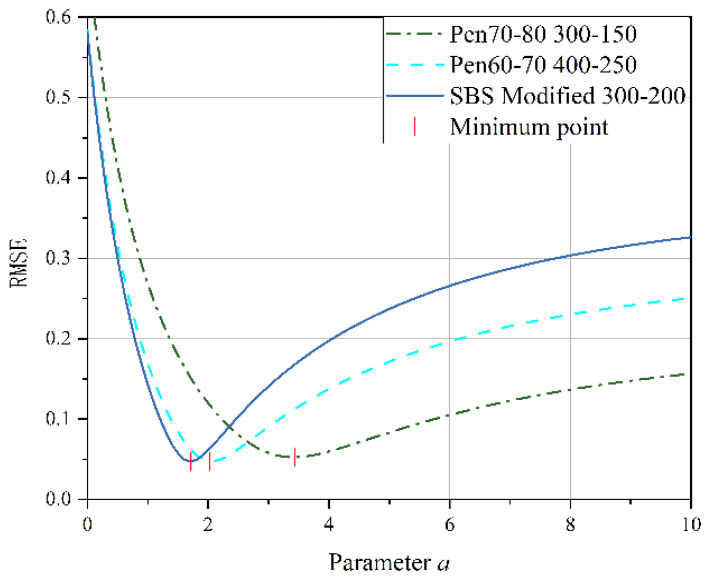
Root mean square error fitting curve of NLVE LVE mode.

In addition to the LVE–NLVE loading mode, this study also conducted time scanning experiments using the NLVE–LVE loading mode for the selected three types of asphalt. Under the NLVE–LVE loading mode, asphalt binders Pen70–80, Pen60–70, and SBS–modified asphalt were subjected to stress combinations of 300–150, 400–250, and 300–200, respectively. The ORZ model was used to predict the second–stage fatigue life of asphalt under this mode and to validate its applicability. Root mean square error curves were generated based on the second–stage damage scores obtained from Table 4, as shown in Figure 12. The y–axis represents the root mean square error, and the x–axis represents the corresponding parameter a. The values of parameter a were determined based on the minimum root mean square error for each loading mode and are listed in Table 7.

**Figure 13:**
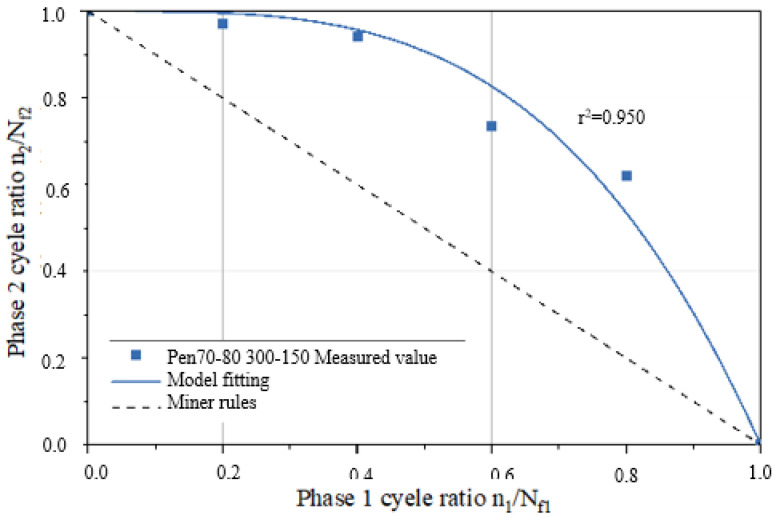
Fitting curve of the second–stage residual life of the Pen70–80 sample.

**Figure 14:**
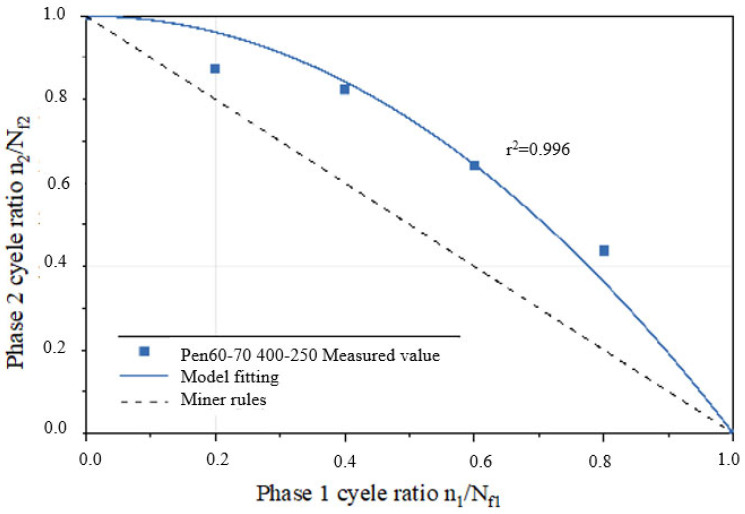
Fitting curve of the second–stage residual life of the Pen60–70 sample.

**Figure 15:**
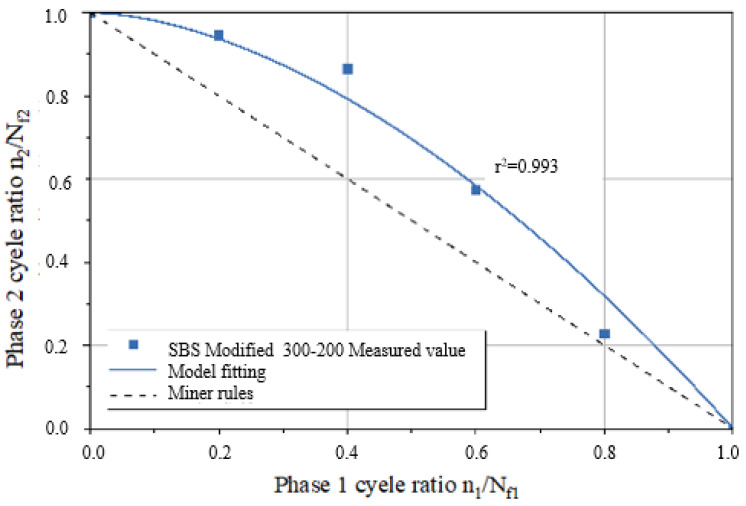
Fitting curve of the second–stage residual life of SBS–modified asphalt sample.

According to the data presented in Table 7, parameter a for the Pen70–80 specimen in the 300–150 loading mode is the highest, followed by the Pen60–70 specimen in the 400–250 loading mode, and the SBS–modified asphalt in the 300–200 variable stress mode has the smallest a value. Analyzing the second–stage residual life curves for the three types of asphalt binders in the NLVE–LVE experiments, as shown in Figure 13, Figure 14 and Figure 15, it is evident that the curve in Figure 13 has a significantly higher curvature compared to Figure 14 and Figure 15, indicating the largest deviation from the Miner criterion predicted values. This corresponds to the largest a value. This suggests that, similar to the LVE–NLVE loading mode, parameter a can also reflect the deviation between the fatigue life observed in the NLVE–LVE fatigue experiments conducted in this study and the Miner criterion predicted values.

Predicted values from the ORZ model were obtained from the three fitting curves and are listed in Table 8. When compared to the experimental measurements, the differences between the predicted and measured values were all less than 0.01. Furthermore, the calculated correlation coefficients (*r*–squared, *r*^2^) for all cases exceeded 0.95, indicating a strong and positive correlation. This demonstrates that the ORZ model can effectively predict the fatigue life of asphalt binder specimens in the NLVE–LVE experiments conducted in this study.

## 4. Conclusions

This study conducted an analysis of rheological parameters such as the complex shear modulus and phase angle obtained from fatigue tests. It compared the differences in the evolution of asphalt sample rheological properties between constant stress–time scanning and variable stress–time scanning. Furthermore, the applicability of the two–stage fatigue life model was verified based on the results obtained from the variable stress scanning tests. The main conclusions drawn from this analysis are as follows:

(1) Under LVE stress, the decay of the complex shear modulus in asphalt binder samples exhibits a short first–stage duration, with the second–stage modulus values remaining relatively constant for a longer duration. In NLVE stress, both Pen70–80 and Pen60–70 asphalt binders experience a degree of modulus recovery, reflecting the self–healing nature of asphalt binders. Most of the modulus decay occurs in the third stage.
(2) Analyzing the complex shear modulus curves obtained from variable stress scanning, it is observed that after NLVE stress, when LVE stress is reapplied, asphalt specimens go through a period of damage recovery. This phenomenon is attributed to the presence of residual stresses caused by NLVE stress cycles. The degree of modulus recovery is negatively correlated with the number of NLVE stress cycles.
(3) Under LVE stress, the dominant type of fatigue damage in the samples is fatigue cracking, resulting in wider crack regions and smaller wavy damage areas. In NLVE stress, the primary form of damage is flow damage, leading to noticeably narrower crack regions, larger wavy damage areas, and a rapid change in damage types over a short period.
(4) After the first–stage LVE stress with a loading cycle number of 0.2*N_f_*_1_, the second–stage remaining fatigue life (*n*_2_) of the samples is consistently greater than *N_f_*_2_, and the second–stage fatigue life increases to different extents compared to when the NLVE stress is applied separately. This phenomenon can be attributed to the “exercise effect” of low stress amplitudes on the samples.
(5) The applicability of the ORZ model was validated by using two loading modes, LVE–NLVE and NLVE–LVE, and parameter *a* was examined. Across various loading modes, the correlation between the fitting curves’ predicted values and experimental measurements was consistently high, confirming the model’s ability to predict the remaining fatigue life of the specimens subjected to the LVE–NLVE and NLVE–LVE fatigue tests conducted in this study.

## Figures and Tables

**Table 1:** Main technical specifications of asphalt binders.

Testing Parameters	Unit	Pen60–70	Pen70–80	SBS	Testing Standards
Penetration Value (25 °C, 100 g, 5 s)	0.1 mm	64	72	56	T0604
Softening Point (TR&B)	°C	48	49	64	T0606
Ductility (10 °C, 5 cm/min)	cm	67	>100	43	T0605
Kinematic Viscosity (135 °C)	Pa·s	0.21	0.34	1.95	T0620
Mass Change of Residue after RTFOT	%	0.102	0.079	0.045	T0609

**Table 2:** Yield stress values for three types of asphalt binders.

Material Types	Pen60–70	Pen70–80	SBS
Yield Stress (kPa)	274	187	229

**Table 3:** Asphalt LVE–NLVE test plan.

Binder Types	Test Number	First–Stage Stress (kPa)	Second–Stage Stress (kPa)	First–Stage Damage Score	Number of Stress Cycles in the Second Stage *N_f_*_2_
Pen70–80	150–300–1	150	300	0.2	Measured value
	150–300–2	150	300	0.4	Measured value
	150–300–3	150	300	0.6	Measured value
	150–300–4	150	300	0.8	Measured value
Pen70–80	300–150–1	300	150	0.2	Measured value
	300–150–2	300	150	0.4	Measured value
	300–150–3	300	150	0.6	Measured value
	300–150–4	300	150	0.8	Measured value
Pen60–70	250–400–1	250	400	0.2	Measured value
	250–400–2	250	400	0.4	Measured value
	250–400–3	250	400	0.6	Measured value
	250–400–4	250	400	0.8	Measured value
SBS modified binder	200–300–1	200	300	0.2	Measured value
	200–300–2	200	300	0.4	Measured value
	200–300–3	200	300	0.6	Measured value
	200–300–4	200	300	0.8	Measured value

**Table 4:** Variable stress scanning test results and cumulative damage statistics.

Varying Stress	First–Stage Damage Score (*n*_1_/*N*_f1_)	Number of Cycles in the First Stage (*n*_1_)	Number of Cycles in the Second Stage (*n*_2_)	Total Number of Cycles (*n*_1_ + *n*_2_)	Second–Stage Damage Score (*n*_2_/*N*_f2_)	*n*_1_/*N*_f1_ + *n*_2_/*N*_f2_
150–300	0.2	1262	890	2152	1.13	1.33
	0.4	2524	860	3384	1.09	1.49
	0.6	3786	585	4371	0.74	1.34
	0.8	5048	315	5363	0.40	1.20
150–400	0.2	1262	400	1662	1.10	1.30
	0.4	2524	350	2874	0.96	1.36
	0.6	3786	265	4051	0.73	1.33
	0.8	5048	205	5253	0.52	1.32
100–300	0.2	7848	795	8643	1.01	1.21
	0.4	15,696	632	16,328	0.80	1.20
	0.6	23,544	420	23,964	0.53	1.13
	0.8	31,392	284	31,676	0.36	1.16
100–400	0.2	7848	405	8253	1.01	1.31
	0.4	15,696	275	15,971	0.80	1.15
	0.6	23,544	240	23,784	0.53	1.26
	0.8	31,392	90	31,482	0.36	1.05

**Table 5:** Parameter *a* values for each loading mode.

Load Mode	Least Root Mean Square Error	Parameter *a*
100–300	0.081	1.826
100–400	0.096	1.715
100–500	0.060	1.343
150–300	0.053	3.465
150–400	0.058	2.983
150–500	0.059	2.020

**Table 6:** Second–stage cycle ratio of LVE–NLVE loading mode.

Number	Measured Value	Predictive Value	Difference	Number	Measured Value	Predictive Value	Difference
100–300–1	1.006	0.957	0.049	150–300–1	1.127	0.996	0.131
100–300–2	0.800	0.830	0.030	150–300–2	1.089	0.960	0.129
100–300–3	0.532	0.626	0.094	150–300–3	0.741	0.829	0.088
100–300–4	0.360	0.348	0.012	150–300–4	0.399	0.534	0.135
100–400–1	1.110	0.937	0.173	150–400–1	1.116	0.994	0.122
100–400–2	0.753	0.792	0.039	150–400–2	0.959	0.936	0.023
100–400–3	0.658	0.587	0.071	150–400–3	0.726	0.788	0.062
100–400–4	0.247	0.319	0.072	150–400–4	0.521	0.492	0.029
100–500–1	0.982	0.929	0.053	150–500–1	0.882	0.962	0.080
100–500–2	0.884	0.775	0.109	150–500–2	0.824	0.846	0.022
100–500–3	0.488	0.568	0.080	150–500–3	0.588	0.645	0.057
100–500–4	0.371	0.304	0.067	150–500–4	0.471	0.364	0.107

Note: the tail numbers 1, 2, 3, and 4 of the test number represent the cycle ratios of 0.2, 0.4, 0.6, and 0.8 in the first stage of the loading mode, respectively.

**Table 7:** Parameter “*a*” values for different asphalt binders under NLVE–LVE loading mode.

Sample Type	Load Mode	Least Root Mean Square Error	Parameter *a*
Pen70–80	300–150	0.055	3.436
Pen60–70	400–250	0.052	2.027
SBS–modified	300–200	0.048	1.716

**Table 8:** Second–stage cycle ratio of NLVE LVE loading mode.

Sample Type	Test Number	Measured Value	Predictive Value	Difference
Pen70–80	300–150–1	0.971	0.999	0.027
	300–150–2	0.941	0.959	0.018
	300–150–3	0.734	0.825	0.091
	300–150–4	0.619	0.536	0.082
Pen60–70	400–250–1	0.875	0.964	0.090
	400–250–2	0.824	0.845	0.021
	400–250–3	0.642	0.645	0.003
	400–250–4	0.438	0.362	0.076
SBS–modified	300–200–1	0.946	0.937	0.008
	300–200–2	0.864	0.793	0.071
	300–200–3	0.574	0.583	0.008
	300–200–4	0.228	0.315	0.087

## Data Availability

The data presented in this study are available on request from the corresponding author.
